# Cloning, expression and transmission-blocking activity of anti-PvWARP, malaria vaccine candidate, in *Anopheles stephensi *mysorensis

**DOI:** 10.1186/1475-2875-9-158

**Published:** 2010-06-11

**Authors:** Saber Gholizadeh, Hamid Reza Basseri, Sedigheh Zakeri, Hossein Ladoni, Navid Dinparast Djadid

**Affiliations:** 1 PhD student of Department of Medical Entomology, School of Public Health, Tehran University of Medical Science, Tehran, Iran; 2 Department of Medical Entomology, School of Public Health, Tehran University of Medical Science, Tehran, Iran, P. O. Box 6446-14155, Tehran, Iran; 3 Malaria and Vector Research Group (MVRG), Biotechnology Research Center (BRC), Pasteur Institute of Iran (PII), Pasteur Avenue, P. O. BOX 1316943551, Tehran, Iran

## Abstract

**Background:**

Notwithstanding progress in recent years, a safe, an effective and affordable malaria vaccine is not available yet. Ookinete-secreted protein, *Plasmodium vivax *von Willebrand factor A domain-related protein (PvWARP), is a candidate for malaria transmission-blocking vaccines (TBVs).

**Methods:**

The PvWARP was expressed in *Escherichia coli *BL21 using the pET-23a vector and was purified using Ni-NTA affinity chromatography from a soluble fraction. Polyclonal antibody was raised against rPvWARP and transmission blocking activity was carried out in an *Anopheles stephensi*-*P. vivax *model.

**Results:**

Expression of full length of PvWARP (minus signal peptide) expression showed a 35-kDa protein. The purified protein was recognized by mouse polyclonal antibody directed against rPvWARP. Sera from the animals displayed significantly a blocking activity in the membrane feeding assay of *An. stephensi *mysorensis.

**Conclusions:**

This is the first report on *P. vivax *WARP expression in *E. coli *that provides an essential base for development of the malaria TBV against *P. vivax*. This may greatly assist in malaria elimination, especially in the oriental corner of WHO Eastern Mediterranean Regional Office (WHO/EMRO) including Afghanistan, Iran and Pakistan.

## Background

*Plasmodium vivax *is responsible for 25-40% of annual cases of malaria especially in parts of Latin America and Asia [1]. Drug resistance in *P. vivax *is spreading and vivax malaria has been considered lethal in a similar way to severe falciparum malaria [1-3], challenging the concept that *P. vivax *malaria as 'benign' [4]. Therefore, the development of new control strategies such as a safe and an effective malaria vaccine is expected to play an important role in controlling *P. vivax *malaria [5].

Based on the life cycle stages, malaria vaccines have focused on candidate target antigens on asexual and sexual stages of the parasite [6]. A transmission-blocking vaccine (TBV) targeting the parasite's sexual stages aims at interfering and/or blocking pathogen development within the vector and halting transmission to the non-infected vertebrate host [7,8]. Most TBV candidate targets have been focused on gametocytes, gametes, zygotes or ookinetes [9]. Li *et al.*, [10] concluded that ookinete micronemal proteins, chitinase 1 (CHT1), circumsporozoite protein/thrombospondin-related anonymous protein-related protein (CTRP) and WARP (von Willebrand factor A domain-related protein) may constitute a general class of malaria TBV candidates. *warp *is expressed in late ookinetes and early oocysts [11,12]. This gene had limited polymorphism in clinical isolates of *Plasmodium falciparum *collected from a wide variety of geographical regions [13]. Similarly, the first large-scale survey, conducted on *pvwarp *polymorphism in both temperate and tropical *P. vivax *field isolates in Iran, showed that there is a limited polymorphism in this gene [14]. Looking to its biological activity, it was shown that mouse polyclonal antisera raised to recombinant PfWARP had nearly a complete (97-100%) transmission-blocking activity of *P. falciparum *to *Anopheles gambiae *mosquitoes [10]. Accordingly, it seems that a parallel study on PvWARP may provide a broader rationale for developing a WARP-based TBV.

*P. vivax *is a dominant malaria parasite outside Africa. In 2008, 11,333 cases of malaria were reported from Iran, which 10,240 (> 90%) were *P. vivax*, 894 *P. falciparum *and the remaining 195 were mixed infection (the Ministry of Health, 2008). On the other hand, *An. stephensi *is an important oriental malaria vector, prevalent in much of the Indo-Pakistan subcontinent and countries around the Persian Gulf [15]. In Sistan and Baluchistan province located in southeastern part of Iran, *An. stephensi* mysorensis is the most dominant biological form [16,17], which readily attacks man and it has been shown that 15.7% were positive for human blood [18-20]. RAPD and rDNA-ITS2 molecular markers revealed that in Iran, *An. stephensi *could be considered as a single species with different biological and ecological forms in various zoogeographical zones [15].

This study aimed to determine the possibility of developing an anti-PvWARP in a *An. stephensi-P. vivax*model that could be applied as a malaria TBV in the oriental corner of EMRO including Afghanistan, Iran and Pakistan. Therefore, this study reports, for the first time, the efficient and successful cloning and expression of full length PvWARP (minus signal peptide), an ookinete-secreted TBV candidate. Recombinant PvWARP elicits potent malaria transmission-blocking antibodies in mice and the blocking of *P. vivax *sporogonic cycle in *An. stephensi *mysorensis was evaluated by these antibodies.

## Methods

### Cloning and sub-cloning of PvWARP

Molecular epidemiology study in the tropical and temperate *P. vivax *isolates revealed that *pvwarp *had a limited polymorphism with two non-synonymous substitution [13]. Therefore, the representative of haplotype II of the Iranian *P. vivax *isolates ([GenBank: FJ170315]) was used as a template for the amplification of full-length PvWARP, amino acid positions 24-295, using 5'...GTG**GGATCC**AAAATAAACCTTGTGTCGC...3' and 5'...ACCC**AAGCTT**GTCCGTAGAGTCGCTGTC...3' as forward (EPvWF) and reverse (EPvWR) primers. The bold sequences show *Bam*HI and *Hind*III restriction sites, respectively. The amplification profile was as follows: denaturation at 95°C for 1 min, followed by 30 cycles of annealing at 60°C for 1 min and extension at 72°C for 1 min with 10 min extra extension time in the last cycle. The amplified fragment was cloned into pGEM-T Easy vector (Promega, USA) after purification from the gel. Selection of the transformed clones was carried out on the Luria-Bertani (LB) agar medium containing 100 μg/ml ampicilin, 1.5 mM isopropyl-β-d-thiogalactopyranoside (IPTG) and 0.04% X-gal. Positive clones were confirmed by plasmid isolation followed by digestion with *EcoR*I and sequencing the cloned fragments. The insert was excised with restriction enzymes *Bam*HI and *Hind*III. After ligation of the insert to the *Bam*HI-*Hind*III sites of vector pET-23a (Qiagen, Germany) containing poly-histidine tag at the C-terminus, the construct, pET-23a+PvWARP was transformed into competent *E. coli *DH5α cells. The recombinant clones were selected on 25 μg/ml chloramphenicol and ampicilin plates. The open reading frame was confirmed by sequencing and this construct was then used to transform *E. coli *BL21 (PlysS) for expression.

### Expression and purification of recombinant PvWARP

The transformed cells were grown overnight in LB medium containing 25 μg/ml chloramphenicol and 100 μg/ml ampicilin with shaking (150 rpm) at 37°C until the OD at 600 nm of culture reached 0.6-0.7. The cells were incubated with 1 mM IPTG (Sigma, USA) and grown at 37°C for four hours . Then they were harvested by centrifugation for 10 min and kept in -80°C until use. The frozen pellet was dissolved in denaturation buffer including 6 M guanidine thiocyanate, 20 mM Tris-HCl, 500 mM NaCl, 20 mM immidazol and 1 mM PMSF, pH 7.9. The cells containing PvWARP inclusion bodies were lysed on ice by 15 cycles of sonication (Ultraschallprozessor; Germany), each cycle consisted of 20 s pulses with 40 s intervals. To remove cellular debris, the bacterial lysate was centrifuged at 10,000 rpm at 4°C for 15 min. The supernatant was incubated with Ni^2+^-nitrilotriacetic acid agarose resin (Ni-NTA Agarose, Qiagen, Germany) at 4°C for 2 h and the resin was transferred into a column. After washing with 6 M urea, 20 mM Tris-HCl, 500 mM NaCl and 30 mM immidazol (pH 7.9), the protein was eluted as 0.5 ml fractions with an elution buffer containing 8 M urea, 50 mM Na_2_HPO_4_, 300 mM NaCl, 250 mM immidazol (pH 7.9). The fractions containing PvWARP were desalted in PBS with Econo-Pac 10DG columns (BioRad, USA) according to the manufacture's manual. All eluates were analyzed by SDS-PAGE on 12% gels under reducing conditions. Finally, the protein concentration was estimated using the Bradford assay. Immunoblotting was carried out by standard protocols, using by both anti-His antibody (Qiagen, Germany) and the sera of mice immunized with rPvWARP.

### Antibody production

Male BALB/c mice (n = 6), six to eight weeks old, were immunized with 50 μg of PvWARP emulsified in complete Freund's adjuvant (CFA) (Sigma, Germany). Then, it was followed by two booster injections (twice at 3-week intervals) with 25 μg of protein emulsified in incomplete Freund's adjuvant. Mice in the control group (n = 6) were immunized with the sera adjuvant formulations in PBS. Pre-immune sera were collected on day 0 as another control group.

### Membrane feeding assay

The antibody activity against development of sexual stage of *P. vivax *was examined using the infected blood of volunteer patients in Sistan and Baluchistan province, Iran where vivax malaria is endemic. First, local *An. stephensi* mysorensis insectarium colony was established in Iranshahr and Chabahar districts, the cities located in southeastern part of Iran. Larvae were reared in bowls at a density of 300 larvae/500 ml of water. The pupae were transferred to 50 × 50 × 50 cm cages made of muslin cloth to harbor adults with free mating. All mosquitoes were maintained at 28 ± 2°C temperature and 70 ± 10% relative humidity with 12:12 h light and dark photoperiods adjusted by fluorescent lighting. During rearing, the adult mosquitoes were fed on 5% glucose solution and the females on rabbit blood. Nulliparous female mosquitoes were starved for 12-18 hours before feeding on a blood meal containing *P. vivax *gametocytes and anti-PvWARP.

Blood containing *P. vivax* gametocytes was collected from local volunteer malaria patients who were attended at local clinics in Chabahar district. In case, the volunteer patients were chosen for interview and in order to avoid any intervention of drug against development of sexual stages of the parasite, only those patients who had not previously taken any anti-malarial drugs for the current infection were chosen as donors. The gametocyte density of the isolate of *P. vivax *was 47 and 75 gametocytes/200 White Blood Cells. In addition, an informed consent was obtained from all individuals who were participated in this study, and an ethical approval was obtained from Pasteur Institute of Iran and Tehran University of Medical Sciences. Then the patients were followed up for treatment by local health services personnel.

Female *An. stephensi *mosquitoes were fed on membrane feeders (constructed from aquarium hitter, beaker and parafilm) containing 200 μl of *P. vivax*-infected blood plus 70 μl of antisera and normal human sera (donor blood group: O+) for 60 min. Non-engorged mosquitoes were removed, and engorged mosquitoes were maintained in double cages with 5% glucose at 28 ± 2°C and 80% relative humidity. Experimental and control groups (PBS+FA, NMS and gametocyte containing blood) were infected in parallel with two independent field isolates of *P. vivax *originated from malaria patients. Mosquito midguts were dissected in PBS 12-14 days after blood meal, stained with mercurochrome 2% and oocysts were enumerated to calculate the transmission blocking activity in different groups.

### Statistical analysis

Statistical analysis was performed using the Mann-Whitney U test to compare differences in geometric mean oocyst density and the proportion of mosquitoes infected between groups was carried out by using SPSS software.

## Results

### Cloning and expression of *pvwarp *fragment

**Figure 1:**
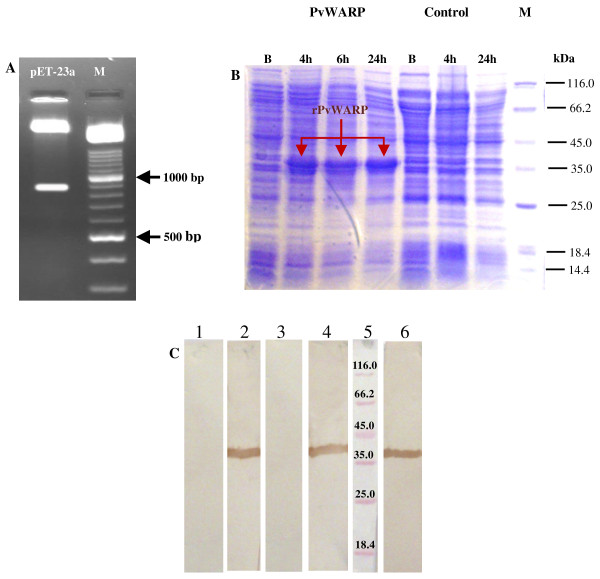
**Cloning, expression and characterization of PvWARP**. **A) **Digestion result of the extracted plasmid (pET-23a) from DH5a by using *BamH*I and *Hind*III restriction enzymes on 1.5% agarose gel. M: DNA molecular marker. **B) **Optimization of PvWARP expression in pET-23a at *E. coli *strain BL21 host in 4, 6 and 24 hours after induction. B: before induction, M: protein molecular weight maker (kDa). **C) **Western blotting of polyclonal antibody for PvWARP. Purified protein was blotted on nitro cellulose membrane and stained by diaminobenzidine; lanes 1: negative control (mice without any protein injection), lane 3 mice immunized with Freund's Adjuvant lane 5, protein size marker; lanes 2, 4 and 6: result of Western Blot in mice immunized with rPvWARP.

The sequence of PvWARP lacking the N-terminal signal sequence amino acid residues 1-23 was amplified from *P. vivax *genomic DNA ([GenBank: FJ170315]) [13]. There were two non-synonymous substitutions at the amino acids T_83 _and R_177 _in comparison with Sal I ([GenBank: XM001608555]), replacing with A and S, respectively. The G/C and A/T contents of *pvwarp *sequence were 48.99% and 51.01%, respectively. Following sub-cloning the fragment into the expression vector pET-23a (Figure 1A), PvWARP was expressed in *E. coli *BL21 cells (Figure 1B). An optimization of the expression in different times was used to yield soluble proteins. We modified expression strategy by growing the cells in LB medium at 37°C and induction with 1 mM IPTG for 4, 6 and 24 h (Figure 1B). Highly efficient induction of a 35-kDa protein was performed at 4 h after induction (Figure 1B). A spot with PvWARP and a molecular weight close to the estimated values calculated for PvWARP (35 kDa) was revealed in SDS-PAGE gel after induction but absent in control (Figure 1B).

### Protein purification and antibody production

The purification protocol was optimized for PvWARP and recombinant protein was purified using Ni-NTA-Agarose beads (Qiagen, Germany) by elusion using imidazole. The yield of purified PvWARP in different independent purifications varied between 300-500 μg/ml of purified solution. Western blot analysis which was carried out with PvWARP showed the recognition of a single band around 35 kDa.

To assess the immunogenicity of purified protein with CFA, six mice were vaccinated with rPvWARP and tested the resultant sera. When a single run of Western blot analysis was carried out, the sera from all mice vaccinated with rPvWARP recognized the immunogen. The sera from control mice vaccinated with adjuvant and non-vaccinated mice showed no antibody response (Figure 1C).

### Transmission blocking assay

**Figure 2:**
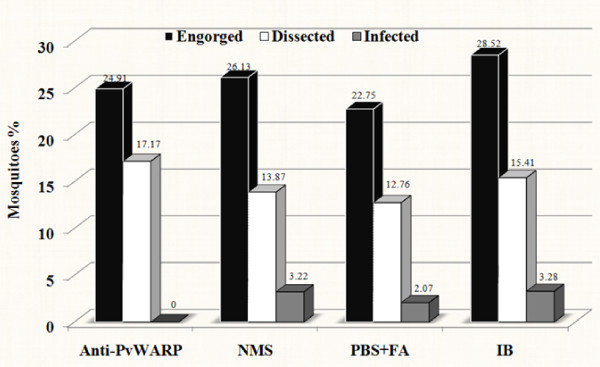
**Inhibitory effect of anti-rPVWARP on infectivity of *An. stephensi *to *P. vivax***. The percentage of engorged, dissected and supporting oocyst in the midgut of *An. stephensi *mysorensis mosquitoes reported as the mean of two different field isolates. NMS: control group mosquitoes fed by infected blood containing normal mouse sera, PBS+FA: control group mosquitoes fed by infected blood containing sera of mouse immunized by PBS and Freund's adjuvant, IB: control group mosquitoes fed only by infected blood.

Membrane feeding experiments were performed to assess the functionality of the immunized sera transmission blocking activity in *An. stephensi *mosquitoes. The result of infecting *Anopheles *were led to the development of *P. vivax *oocysts only in midgut of mosquitoes artificially fed on NMS, PBS+FA and infected blood (Figures 2). The ratios of mosquitoes supporting oocyst in their midgut were about 21-23% in control group that fed on gametocyte-infected blood from field isolates. The presence of oocyst in controls including PBS+FA and NMS was about 16% to 21%. Antisera to rPvWARP showed a strong reduction in the number of oocysts (*P *< 0.0001). However, *An. stephensi *mosquitoes fed with *P. vivax *gametocyte-infected blood containing anti-rPWARP developed no oocyst in their midgut. Geometric mean oocyst numbers per midgut in different control groups ranged between 1.15 and 1.44 (Table 1).

**Table 1:** Effect of anti-rPvWARP sera raised in mouse on *P. vivax *infectivity to *An. stephensi *mosquitoes

Experiment	Antibody	Geometric mean no. of oocysts in midgut (range)	No. infected/total engorged mosquitoes (% prevalence)
1	Control (Infected blood)	1.43 (0-3)	5/23 (21.74%)
	Control (PBS+FA)	1.15 (0-2)	3/18 (16.6%)
	Control (NMS)	1.26 (0-2)	5/24 (20.8%)
	Anti-PVWARP	0.00 (0-0)	0/28 (0%)

2	Control (Infected blood)	1.43 (0-3)	5/22 (22.73%)
	Control (PBS+FA)	1.19 (0-3)	3/19 (15.8%)
	Control (NMS)	1.44 (0-2)	5/19 (21.05%)
	Anti-PVWARP	0.00 (0-0)	0/23 (0%)

## Discussion

The current study reports, for the first time, the cloning and expression of rPvWARP, ookinete-secreted protein from field collected *P. vivax *isolates. The anti-recombinant protein (anti-PvWARP), which is produced in the mice immunized with Freund's adjuvant formulation, showed transmission-blocking activity.

Continuous reduction of malaria transmission by using vaccines targeting sexual stage in malaria transmission will be essential for achieving the global action plan goal of Roll Back Malaria, elimination and perhaps eradication of this disease from various endemic countries. To assess transmission blocking activity, all previous studies were carried out in African malaria vector, *An. gambiae *and human or other non-human malaria parasites. However, in this study, *An. stephensi-P. vivax *model was employed to study the efficacy of raised antibody against PvWARP over field conditions. *An. stephensi *is the major vector of human malaria outside Africa (Middle East and South Asia), and belongs to the same subgenus as *Anopheles gambiae*. On the other hand, *P. vivax *as a neglected parasite is predominant cause of human malaria in the Middle East, central and southeast Asia and South America [21].

In addition, results of parasite development in the current study is in agreement with report of Basseri *et al. *[17] about the competency of *An. stephensi mysorensis *strain to *P. vivax *in the same area that from all infected mosquitoes with *P. vivax*, the overall number of mosquitoes supporting oocysts in their midgut was 21.9%. In this study, 15.8-22.73% of the infected mosquitoes in the different control groups (NMS, PBS+FA and Infected Blood) developed the oocyst in their midguts. It seems that the low number of developed oocyst on midgut of female mosquitoes is a bottleneck for life cycle of *Plasmodium *parasites, which may provide more chance for inhibiting the parasite development using TBV, though drawing any conclusion needs more robust data and further field experiments.

## Conclusions

Antigens such as WARP, which is expressed on the surface of mosquito-stage parasites have been postulated as TBV candidates because it seem not to be under the selective pressure mediated by the vertebrate immune system. Consequently, this protein exhibited low levels of polymorphisms favouring the efficacy of vaccines derived from a unique antigenic variant. Further, as a certain types of adjuvants induce reactogenicity in humans [22] and animal models, the use of other adjuvants to boost TBV antigen response is a significant issue to be resolved. Although anti-malarial drugs currently work well against vivax malaria, a vaccine that targets *P. vivax *would probably reduce the transmission rate and parasite density within local populations which may greatly assist in complete malaria elimination, especially in this oriental corner of EMRO including Afghanistan, Iran and Pakistan.

## Competing interests

The authors declare that they have no competing interests.

## Authors' contributions

SG carried out the cloning and expression studies in lab, transmission blocking assay in the field and drafted the manuscript. HRB and NDD designed the study, supervised the lab, field work, and jointly finalized the manuscript. SZ advised in lab and field practical and participated in finalizing the manuscript. HL contributed in data collection and administrative coordination. All authors read and approved the final manuscript.
